# Validation of the Persian Version of the 8-Item Morisky Medication Adherence Scale (MMAS-8) in Iranian Hypertensive Patients

**DOI:** 10.5539/gjhs.v7n4p173

**Published:** 2014-12-31

**Authors:** Yashar Moharamzad, Habibollah Saadat, Babak Nakhjavan Shahraki, Alireza Rai, Zahra Saadat, Hossein Aerab-Sheibani, Mohammad Mehdi Naghizadeh, Donald E. Morisky

**Affiliations:** 1 Kermanshah University of Medical Sciences, Kermanshah, Iran; 2 Cardiovascular Research Center, Shahid Beheshti University of Medical Sciences, Tehran, Iran; 3 Private Practice, Zahedan and Karaj, Iran; 4 Cardiology Department, School of Medicine, Kermanshah University of Medical Sciences, Kermanshah, Iran; 5 Shahid Sadoughi University of Medical Sciences, Bafgh, Yazd, Iran; 6 Community Medicine Group, Faculty of Medicine, Fasa University of Medical Sciences, Fasa, Iran; 7 Department of Community Health Sciences, UCLA Fielding School of Public Health, Los Angeles, USA

**Keywords:** Persian, Iran, validity, reliability, Morisky Medication Adherence Scale (MMAS), hypertension, anti-hypertensive adherence

## Abstract

The reliability and validity of the 8-item Morisky Medication Adherence Scale (MMAS-8) was assessed in a sample of Iranian hypertensive patients. In this multi-center study which lasted from August to October 2014, a total of 200 patients who were suffering from hypertension (HTN) and were taking anti-hypertensive medication(s) were included. The cases were accessed through private and university health centers in the cities of Tehran, Karaj, Kermanshah, and Bafgh in Iran and were interviewed face-to-face by the research team. The validated Persian translation of the MMAS-8 was provided by the owner of this scale. This scale contains 7 questions with “Yes” or “No” response choices and an additional Likert-type question (totally 8 questions). The total score ranges from 0 to 8 with higher scores reflecting better medication adherence. Mean (±SD) overall MMAS-8 score was 5.57 (±1.86). There were 108 (54%), 62 (31%), and 30 (15%) patients in the low, moderate, and high adherence groups. Internal consistency was acceptable with an overall Cronbach’s α coefficient of 0.697 and test–retest reliability showed good reproducibility (r= 0.940); P< 0.001. Overall score of the MMAS-8 was significantly correlated with systolic BP (r= - 0.306) and diastolic BP (r= - 0.279) with P< 0.001 for both BP measurements. The Chi-square test showed a significant relationship between adherence level and BP control (P= 0.016). The sensitivity, specificity, positive predictive value (PPV), and negative predictive value (NPV) of the scale were 92.8%, 22.3%, 52.9%, and 76.7%, respectively. The Persian version of the MMAS had acceptable reliability and validity in Iranian hypertensive patients. This scale can be used as a standard and reliable tool in future studies to determine medication adherence of Persian-speaking patients with chronic conditions.

## 1. Introduction

Hypertension (HTN) is a major public health issue worldwide. If uncontrolled with adequate and appropriate medication(s), it imposes serious health problems on sufferers such as heart attack, heart failure, stroke, renal failure, etc. in long-term run (Krousel-Wood, Muntner, Islam, Morisky, & Webber, 2009). Currently, there are effective medications available on the pharmaceutical market to control blood pressure (BP) of patients sufficiently. In spite of availability of these therapeutic agents, studies show that many patients who are taking anti-hypertensives do not meet the criteria for controlled BP within defined target limits (Ong, Cheung, Man, Lau, & Lam, 2007). Quiet similar to other communities, previous reports from Iran have documented uncontrolled BP in 62% (Arabzadeh et al., 2014) to 65% (Ebrahimi et al., 2006) of HTN patients, which obviously are significant numbers to be considered. Good adherence to (compliance with) anti-hypertensive medications by patients is one of the main key factors to succeed in controlling high BP and minimizing the future risks of HTN complications, hospitalizations, disabilities, and related financial burden on healthcare systems (Krousel-Wood et al., 2009; Pittman, Tao, Chen, & Stettin, 2010).

The definition of adherence is the extent to which a patient takes his/her prescribed medication(s) following the instructions provided by doctor (Osterberg & Blaschke, 2005). Measuring the adherence of patients could be a challenging problem for clinicians. There are different tools to determine adherence to medications. One of the reliable and widely used scales in this regard is the 8-item Morisky Medication Adherence Scale (MMAS-8) (Morisky, Ang, Krousel-Wood, & Ward, 2008). The efforts to develop this scale started in 1975, and then in 1986 a 4-item scale was introduced by the developer. This 4-item scale was then revised and updated in 2008, based on focus group discussions and feedbacks from several studies, to additionally encompass the adherence behavior of the respondents. As a result of this update, the current MMAS-8 was developed as a simple and reliable tool which can be used by clinicians to determine the adherence of patients to prescribed medications (Morisky & DiMatteo, 2011). The 8-item scale was originally studied in hypertensive patients and the results revealed that it was a reliable (α= 0.83) tool and showed significant correlation with BP control (P< 0.05). It showed a sensitivity of 93% in detecting patients with poor BP control (Morisky et al., 2008). Since its introduction, the MMAS-8 has been studied in different conditions and languages including French (Korb-Savoldelli et al., 2012), Portuguese (de Oliveira-Filho, Morisky, Neves, Costa, & de Lyra, 2014), Turkish (Hacıhasanoğlu Aşılar, Gözüm, Capık, & Morisky, 2014), Arabic (Alhewiti, 2014), Urdu (Saleem et al., 2012), Chinese (Yan et al., 2014), Malay (Al-Qazaz et al., 2010), Taiwanese and Mandarin (Lin et al., 2013), etc.

Considering the aforementioned facts with regard to uncontrolled HTN in Iranian patients and the key role of good adherence to anti-hypertensives, having a reliable, handy, and simple to calculate tool to determine adherence seems necessary for Iranian clinicians and researchers. The reliability and validity of the MMAS-8, as per a meticulous review of both English and Persian literature, has not been investigated in Iranian or other Persian-speaking populations to date. Hence, we decided to carry out this study to determine the reliability and validity of the Persian translation of the MMAS-8 in a sample of Iranian hypertensive patients. If the results will reveal satisfactory reliability and validity of this scale among Persian-speaking individuals, it can be used as a standard and accurate tool in the future studies by other researchers in studies addressing medication compliance in the respective population.

## 2. Materials and Methods

### 2.1 Setting and Participants

This cross-sectional study lasted from August to October 2014. This was a multicenter study including cardiology clinic of university hospital, private cardiology office, pharmacy, and private general practitioner office in the cities of Tehran, Karaj, Kermanshah, and Bafgh. Inclusion criteria were adult patients of either gender who had documented hypertension (either on medical records or self-reported) for the past 6 months and were taking anti-high blood pressure medications. The patients were interviewed directly (face-to-face) upon their presentation for checking their BP or to refill their prescriptions or any other complaint. Firstly, the patients were instructed about the scale and then were asked to fill out the scale. The patient was allowed to accept or refuse participating at the study. If the patient was illiterate, then the researcher read the items of the scale for patient and asked him/her to respond to them orally and then the answer was inserted on the form by the researcher. If the patient had a medical record in the center, his/her record was checked by the researcher to assure the accuracy of data provided by the patient, in particular the duration of hypertension, medications prescribed, and other comorbidities. If the patient was a new patient to the clinics and no documented record was available, we relied solely on patient’s statements about his/her condition, its duration, and medications used.

### 2.2 Instruments

The validated Persian translation of the MMAS-8 was provided by Prof. Donald E. Morisky, the owner of this scale, as well as permission to use the scale in this study. The translation to Persian was done by an international linguistic organization which provides services to global healthcare systems. This institute has collaboration with the European Medicines Agency. The translation protocol of the MMAS-8 is outlined in Appendix 1.

The MMAS-8 has 8 items (Appendix 2). Response choices for items 1 to 7 are “Yes” or “No”. The question No. 8 is a Likert-type question. The total score ranges from 0 to 8. Scores of less than 6 indicate low adherence, scores of of 6 to < 8 indicate moderate adherence, and score = 8 indicates high adherence.

In addition to the MMAS-8, a checklist was designed by the authors after pertinent literature review to gather demographic as well as variables about other diseases or medications the patients were taking. First, the MMAS-8 was completed by the patients. Then, the checklist data was completed. The data included in the checklist were demographic data (age, gender, weight, height, occupation, educational level), duration of hypertension, medications prescribed for hypertension, awareness of the patient about his/her current blood pressure, other comorbidities, other medications other than anti-hypertensives, and control of hypertension during the last 6 months by a healthcare provider. Following the survey, the blood pressure of the patients was measured by the researchers using a sphygmomanometer on the left arm when the patient was in seated position. The patients were asked to seat relaxed and not smoke for half an hour before blood pressure recording. Korotkoff sounds were the basis to define systolic and diastolic blood pressure.

### 2.3 Statistics

*Sample size calculation*

At first, a pilot study including 25 patients was done to yield the required sample size. The pilot showed that mean (±SD) of the MMAS was 5.7 (±1.7). Based on sample size formulation for quantitative studies with 95% confidence (alpha= 0.05) and a standard deviation of 1.7 for detecting a 0.25 unit difference of mean, we needed a minimum number of 178 patients.

*Internal consistency reliability analysis*

The internal consistency for each item of the scale as well as the scale itself was assessed by calculating Cronbach’s α coefficient. This coefficient indicates whether or not each item in a scale is appropriate for determining the underlying concept of the scale addressed. The higher the coefficient, the more consistent is the questionnaire. Generally, values calculated to be equal or higher than 0.5 are regarded to indicate satisfactory internal consistency; 0.7 and 0.8 are good, 0.8 and 0.9 are great, and > 0.9 are superb (Parsian & Dunning, 2009). Herein, the Cronbach’s α was set at 0.5.

*Construct validity*

To determine the construct validity of the scale, which addresses how items in the scale are related to the relevant theoretical construct (Parsian & Dunning, 2009), factor analysis of the collected data was used. Before conducting factor analysis, Kaiser-Meyer-Olkin (KMO) and Bartlett’s tests were used to determine sufficient sample size and its suitability for factor analysis. The construct validity of the questionnaire was analyzed by a principal component analysis (PCA) with varimax rotation. The number of components to be retained in the PCA was examined using the Horn’s parallel analysis (1000 iterations) and confirmatory factor analysis.

*Test–retest reliability*

Retest reliability was calculated to determine stability of the scale. The researcher expects that with re-administration of a test to the same sample after for example two weeks, there will be no substantial change in the responses provided by the sample. In other words, retest reliability inspects the probability of a measure to yield the same description of a given variable if that measure is repeated (Horne, Hankins, & Jenkins, 2001; Parsian & Dunning, 2009). Pearson’s correlation coefficient r scores range between -1 and +1: magnitudes of +1 show highest correspondence and 0 shows no correspondence. Instruments showing r values greater than 0.80 are considered to be very reliable; however, the reliability also depends on the expected stability of the construct being measured (de Oliveira-Filho et al., 2014). Test–retest reliability was assessed through the administration of a second MMAS-8 to a random sample of 32 patients who were contacted and visited for the second time 14 days after the initial visit. The same interviewer carried out the test and retest interviews.

*Known groups’ validity (criterion-related validity)*

Known groups’ validity can be assessed by testing the ability of a measure to distinguish between groups of individuals that differ from each other considering a known factor (de Oliveira-Filho et al., 2014). Here, known groups’ validity was assessed through investigating the association between controlled BP (i.e., systolic BP < 140 mmHg and diastolic < 90 mmHg) and the MMAS-8 categories (i.e., low, medium, and high adherence) using the Chi-squared and analysis of variance (ANOVA) tests followed by the Tukey test. We expected that those who scored lower on the MMAS-8, literally translated to lower adherence level, were more likely to have uncontrolled BP (Morisky et al., 2008). P values of less than 0.05 were considered statistically significant.

*Sensitivity and specificity*

In order to answer to this question that how well the studied Persian version of the MMAS-8 would be helpful in identifying patients with poor BP control, sensitivity, specificity, positive predictive value (PPV) and negative predictive value (NPV) were calculated through a dichotomous low/moderate adherence vs. high adherence subjects.

*Statistical analysis*

Data are presented as mean (±standard deviation, SD) and frequency (percentage). Statistical analyzes involved the Chi-square and ANOVA tests followed by the Tukey post-hoc test to test the association between adherence and other independent variables (age, gender, educational level, and controlled BP control). BP under control was defined as systolic BP values < 140 and diastolic BP < 90 mm Hg. The significance level was set as P < 0.05. Data analysis was performed using the SPSS software for Windows (ver. 18.0) (SPSS Inc, Chicago, IL).

### 2.4 Ethics

Since no therapeutic or diagnostic intervention was done in this study, we gave instructions to the patients orally before completing the MMAS-8 and the checklist. After that, if agreed by the patient, oral consent was obtained. They were assured that the information they provide will be used just for scientific purposes and will not be disclosed to other persons or organizations. The study protocol was in conformity with the ethical guidelines of the 1975 Declaration of Helsinki.

## 3. Results

*Socio-demographic data and hypertension history*

A total of 200 patients completed the MMAS-8. Mean (±SD) age of the cases was 59.7 (±27.2) years (range, 39-86 years) and 80% of the sample was older than 50 years of age. There were 84 men (42%). Most of them (84.5%) were under coverage of health insurance services. Forty cases (20%) were current cigarette smokers. Table 1 depicts demographic characteristics of the patients.

**Table 1:** Demographic characteristics of 200 hypertensive patients

Variable		Frequency (percentage)
Gender	Female	116 (58%)
	Male	84 (42%)
Body mass index, Kg/m^2^	< 26	76 (38%)
	≥ 26	124 (62%)
Educational level	Illiterate	47 (23.5%)
	Lower than high school diploma	64 (32%)
	High school diploma	60 (30%)
	Academic degrees	29 (14.5%)
Occupation	Market/self-employed	53 (26.5%)
	Clerk	31 (15.5%)
	Housewife	91 (45.5%)
	Retired	25 (12.5%)

Mean (±SD) duration passed from diagnosis of hypertension was 7.2 (±5.69) years. About 81.5% of the patients stated that they had scheduled appointments with their doctors to have their BP checked during the last 6 months. About 33% (66 cases) gave history of being observed in emergency services due to sudden increase in their BP (hypertensive crisis). In Table 2 more details about variables related to hypertension are presented.

**Table 2:** Variables related to hypertension in 200 Iranian patients who were under treatment with anti-hypertensives

Variable			
Physician		GP	57 (28.5%)
		Internist	23 (11.5%)
		Cardiologist	53 (26.5%)
		Nephrologist	5 (2.5%)
		More than one doctor	62 (31%)
Medication	Monotherapy	ARB	57 (28.5%)
		ACEI	10 (5%)
		SBB	19 (9.5%)
		Hydrochlorothiazide	11 (5.5%)
	Combination therapy*	103 (51.5%)	
Comorbidity		119 (59.5%)	
Self-awareness of BP value		124 (62%)	
Correct awareness		62 (31%)	
Incorrect awareness/no awareness		138 (69%)	
Self-awareness of systolic BP	Not aware	76 (38%)	
	Faulty awareness	46 (23%)	
	Correct awareness	78 (39%)	
	Not aware	76 (38%)	
	Faulty awareness	39 (19.5%)	
	Correct awareness	85 (42.5%)	
Self-measured systolic BP, mmHg		140.40 (±19.42)	
Self-measured diastolic BP, mmHg		88.48 (±14.59)	
Physician-measured systolic BP, mmHg		135.9 (±15.95)	
Physician-measured diastolic BP, mmHg		84.13 (±9.55)	

Abbreviations: BP=blood pressure; GP=general practitioner; ARB=angiotensin receptor blocker; ACEI=angiotensin-converting enzyme inhibitor; SBB=selective beta blocker.

* Combination therapy included combination of various anti-hypertensive classes not just limited to those outlined as monotherapy including calcium-channel blocker and other classes of diuretics.

*Internal consistency*

Overall Cronbach’s α coefficient was 0.697 for the 8 items of the Persian version of the MMAS-8. The highest item-to-total correlation coefficient was 0.644 for item 8. The lowest item-to-total correlation coefficients were 0.257 for item 2 and 0.293 for item 7, although significantly different from zero. The Cronbach’s alpha values if item deleted were lower than the resulting coefficient in each item, indicating that the exclusion of items does not affect to increase reliability of the instrument (Table 3).

**Table 3:** Corrected item-to-total correlation and factors loading in principal component analysis (PCA)

	Patients’ responses	Answers			Corrected item-total correlation	Cronbach’s alpha if item deleted	Loading factors

		No. (%)	Mean	SD			
Question 1	No	119 (59.5%)	0.59	0.49	0.419	0.662	0.224
Question 2	No	164 (82.0%)	0.82	0.39	0.257	0.693	0.430
Question 3	No	140 (70.0%)	0.70	0.46	0.432	0.660	0.401
Question 4	No	125 (62.5%)	0.63	0.49	0.379	0.670	0.184
Question 5	Yes	174 (87.0%)	0.87	0.34	0.315	0.685	0.776
Question 6	No	125 (62.5%)	0.63	0.49	0.426	0.660	0.648
Question 7	No	128 (64.0%)	0.64	0.48	0.293	0.687	0.146
Question 8	Never	48 (24.0%)					
	Rarely	74 (37.0%)					
	Sometimes	65 (32.5%)					
	Usually	13 (6.5%)					
	All the time	0 (0.0%)					

Overall Cronbach’s alpha for 8 items= 0.697.

*Anti-hypertensive adherence*

Mean (±SD) overall MMAS-8 score was 5.57 (±1.86). More than half of the patients (108 cases, 54%) were in the low adherence group (i.e., MMAS-8 score < 6). Sixty-two patients 62 (31%) were moderate adherers (i.e., MMAS-8 score= 6 to <8), and 30 (15%) were high adherers (i.e., MMAS-8 score= 8). Patients had best adherence in answer to question 5 (Did you take your antihypertensive medicine yesterday?) with 87% responded “Yes” and had worst adherence in answering to the question 1 (Do you sometimes forget to take your antihypertensive pills?) with just 59.5% answered “No” (Table 3).

*Construct validity analysis*

KMO value was determined as 0.748, which was observed to be a suitable value for the analysis of essential variables. Similarly, Barlett’s test results (χ2=244.4, P<0.001) suggested that data were inter-related and suitable for factor analysis.

The PCA with varimax rotation indicated that the two component accounts for 60.6% of variance in the dataset (32.6% for the first components). Five items had factor loadings > 0.4 (items 2, 3, 5, 6, and 8) on the first component in the PCA presented in Table 1. Item 5 had the highest correlation with the first component of the PCA (r= 0.77), followed by item 8 (r= 0.71). On the second component, three items (items 1, 4, and 7) were extracted. Item 7 had the highest correlation with the second component of the PCA (r= 0.81).

*Test–retest reliability*

The test–retest reliability of the Persian version of the MMAS-8 showed satisfactory reliability and stability of the instrument with Spearman’s rank correlation coefficient of 0.940 (P< 0.001).

*Known groups’ validity*

To find relationship between poor BP control and lower levels of adherence to medications administered, analyses conducted showed that just 4.5% of the patients with hypertensive crisis experience had full adherence to medications. This was significantly lower than that of patients without such crisis experience (P< 0.001).

**Figure 1:**
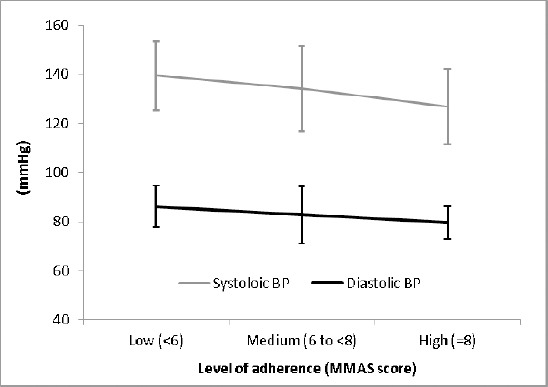
Systolic and diastolic blood pressure changes at different levels of medication adherence

Overall score of the MMAS-8 was significantly correlated with systolic BP (r= - 0.306) and diastolic BP (r= - 0.279) with P< 0.001 for both BP measurements. Mean (±SD) systolic BP in high adherence patients was 126.8 (±15.4) mmHg which was significantly lower than that of patients with medium level of adherence (134.0±17.4 mmHg) (P= 0.017) and low adherers (139.5± 14.1 mmHg); P< 0.001. Also, mean (±SD) diastolic BP in low adherence patients was 82.2 (±8.4) which was higher than patients at moderate adherence level (82.7±11.5 mmHg) (P= 0.057) and significantly higher than those who were high adherers (79.7±6.7 mmHg); P= 0.001. (Figure 1).

In Table 4, validity of the MMAS-8 was compared between those who reported correct awareness of their BP measurement vs. those who were not aware or provided an incorrect report.

**Table 4:** Known groups’ validity considering awareness of patients about their blood pressure value and having a previous history of hypertension crisis

		Adherence level according to (MMAS score)			

		Low (< 6) (N= 108)	Moderate (6 to < 8) (N= 62)	High (=8) (N= 30)	P value
Correct self-awareness of BP	Yes	30 (48.4%)	20 (32.3%)	12 (19.4%)	0.426
	No	78 (56.5%)	42 (30.4%)	18 (13.0%)	
Self-awareness of BP (correct and incorrect)	No	41 (53.9%)	25 (32.9%)	10 (13.2%)	0.811
	Yes	67 (54.0%)	37 (29.8%)	20 (16.1%)	
Hypertensive crisis	No	59 (44.0%)	48 (35.8%)	27 (20.1%)	< 0.001
	Yes	49 (74.2%)	14 (21.2%)	3 (4.5%)	

The Chi-square test showed a significant relationship between adherence level and BP control (P= 0.016), as 76.7% of the high adherence patients had controlled BP, while 54.8% and 42.6% of those in the medium and low adherence groups had controlled BP, respectively (Table 5).

**Table 5:** Relationship between level of anti-hypertensive adherence and blood pressure under control

Blood Pressure (BP)	Adherence level according to (MMAS score)			

	Low (< 6) (N= 108 patients)	Moderate (6 to <8) (N= 62 patients)	High (= 8) (N= 30 patients)	P value
Controlled BP (N= 103, 51.5%)	46 (42.6%)	34 (54.8%)	23 (76.7%)	0.003
Uncontrolled BP (N= 97, 48.5%)	62 (57.4%)	28 (45.2%)	7 (23.3%)	

Controlled blood pressure= Systolic BP< 140 mmHg and diastolic< 90 mmHg. Sensitivity= [(62 + 28)/(62+28+7)]×100= 92.8. Specificity= [23/(46+34+23)]×100= 22.3. Positive predictive value (PPV)= [(62+28)/(62+28+46+34)]×100= 52.9. Negative predictive value (NPV)= [23/(23+7)]×100= 76.7

*Sensitivity and specificity*

As stated earlier, the patients were divided into two groups (low and moderate adherence together as one group vs. high adherers). Using a cut-point of less than 8, the sensitivity and specificity of the scale to identify patients with poor BP control were respectively 92.8% and 22.3%. PPV and NPV were respectively 52.9% and 76.7% (Table 5).

## 4. Discussion

This study aimed to validate the Persian version of the MMAS-8. Generally speaking, the findings obtained are promising and indicate that the translated version of the MMAS-8 studied here had acceptable reliability in Iranian patients with hypertension. As far as we know, there has been no study in the past to validate specific scale to measure medication adherence in Iranian patients. In comparison to the original MMAS-8 which reported Cronbach’s α of 0.83 (Morisky et al., 2008), the estimated reliability here was lower (α= 0.697). This could be the result of differences in characteristics of the two studies and lower sample size here (200 patients) compared with 1400 patients studied in the original MMAS-8 psychometrics evaluation. Considering the fact that sample size can affect internal consistency, this discrepancy may, at least in some part, justified by this factor (Yan et al., 2014). The reference study (Morisky et al., 2008) was performed in one teaching hospital, but here we recruited the patients from different settings with presumption that cultural differences at different geographical areas of the country may be substantial enough to avoid us from reaching a comprehensive conclusion. The reported α values by previous studies in different countries include 0.54 from France employing 199 patients (Korb-Savoldelli et al., 2012), 0.682 from Brazil employing 937 patients (de Oliveira-Filho et al., 2014), 0.70 from Pakistan recruiting 150 patients (Saleem et al., 2012), 0.77 from China studying 176 patients with myocardial infarction (Yan et al., 2014), and 0.79 from Saudi Arabia (Alhewiti, 2014) and Turkey (Hacıhasanoğlu et al., 2014). Compatible with previous reports (Yan et al., 2014; Al-Qazaz et al., 2010), here we found good reproducibility of the Persian version of the MMAS-8.

Regarding the known groups’ comparison the results showed that the Persian MMAS-8 was valid enough to discriminate patients with poor and good blood pressure control. It was valid in distinguishing both controlled and uncontrolled systolic and diastolic BP measurements with statistically significant differences between controlled and uncontrolled BP. This supports that construct validity of the scale is acceptable. This finding is in total agreement with a similar previous report investigating the Portuguese version of the MMAS-8 In a previous study (Oliveira-Filho, Barreto-Filho, Neves, & Lyra Junior, 2012).

The strength of this study was that it was performed in several medical centers including university affiliated tertiary heart center, private cardiology office, private general practitioner office, and pharmacy at different parts of Iran. This enabled us to access a heterogeneous sample of patients with different cultures. Karaj, located about 30 km from the capital, Tehran, has faced an increasing growth in its population during the last 2 decades. Most current residents of this 2-million population city are immigrants from other cities throughout the country. Therefore, we think that the findings here are fairly generalizable to Iranian population.

The limitation here was that in some patients we did not have medical records. They presented to the pharmacy to take their medication or it was the first time that they came to receive health services at a specific center where sampling was done. So the information obtained about duration of hypertension and other medications used as well as other diseases are solely based on subjective information the patients provided to us. So, it is likely that some parts of information are not completely exact and especially recall bias may be a limitation. However, this was not the case in all patients. In about half of the studied patients they had medical records and were known cases of hypertension and the accuracy of the data gathered was confirmed by the researchers interviewed them. Another limitation was dealing with some of the patients who were illiterate. At first we decided to exclude these patients, but since the number of patients was considerable, and since we decided that the results to be as similar as possible to real life practice physicians encounter in every day clinical practice, we decided not to exclude these patients. Another limitation is related to blood pressure recording. Since this study was done in different centers and blood pressure recording was based on measurement by sphygmomanometer and taken by different physicians, it is likely that there might be variations in devices and maybe some variations, though we think not very significant, differences in blood pressure readings. Our resources and limited time did not allow us to implement more accurate devices for blood pressure monitoring such as Holter monitor.

## 5. Conclusion

The Persian version of the MMAS had acceptable reliability and validity in Iranian hypertensive patients. We suggest the MMAS-8, as a reliable and valid tool for Persian-speaking patients, to cardiologists in other cardiac conditions as well as physicians and researchers in other disciplines who are willing to study medication adherence in other chronic conditions which necessitate long-term taking of medication(s) by the suffering patient.

## Appendix 1. Translation Protocol for MMAS Translation in Foreign Languages

**Step 1: Concept elaboration**: The agency project manager develops a concept elaboration document, which describes the intentions of each question in the scale and offers definitions of key words and terms. This aids translators in choosing the appropriate wording in the target language. This report is typically reviewed by the instrument developer prior to being sent to translators.

**Step 2: Forward translations**: The source scale is translated by two translators (T1 & T2). The translators are both native speakers of the target language or qualified to translate into that language by a creditable institution (here, MAPI Institute). The translators work independently of each other.

**Step 3: Reconciliation**: The first translator (T1) combines the two forward translations into a third translation (T3), to maximize harmonization with the source document.

**Step 4: Back translations**: The reconciled translation (T3), is translated back into English by two translators (T4 & T5). The translators are both native speakers of English or qualified to translate into English by a creditable institution (here, MAPI Institute). The translators work independently of each other and work with no prior knowledge of the source version.

**Step 5: Back translation review**: The Oxford Outcomes project manager reviews the back translations (T4 & T5) against the source document and works with the first translator (T1) to a) refine the translation (T6) where necessary and b) clarify any ambiguities arising from the back translations.

**Step 6: Developer review**: The instrument developer reviews the back translation review. Any questions or comments are reviewed by the first translator (T1) and the project manager, and discussions continue until all parties are satisfied with the outcome (T7).

**Step 7: Cognitive debriefing (pilot-testing):** The first translator (T1) recruits five patients in the target population and asks them to complete a copy of the translated scale (T7). After they have completed the scale, the subjects are asked a series of questions aimed at gauging their understanding of the translation. Any issues are discussed between the translator (T1) and the project manager until resolved (T8).

**Step 8: The final translation (T8)** is formatted in the preferred format of the client/developer and sent to two proofreaders. The proofreaders work sequentially and independently. Both proofreaders are native speakers of the target language and are briefed to not suggest changes which invalidate the work done at steps before, i.e. they only should point out spelling mistakes (or similar) rather than make stylistic or preference-based changes.

**Step 9: Step 8 results in the final translation**, which is sent to the client and developer who reviews each item in the scale for its face and construct validity.

## Appendix 2. The 8-item Morisky Medication Adherence Scale (MMAS-8)

Item 1. Do you sometimes forget to take your antihypertensive pills?

Item 2. People sometimes miss taking their medications for reasons other than forgetting. Thinking over the past 2 weeks, were there any days when you did not take your antihypertensive medicine?

Item 3. Have you ever cut back or stopped taking your medication without telling your doctor, because you felt worse when you took it?

Item 4. When you travel or leave home, do you sometimes forget to bring along your antihypertensive medication?

Item 5. Did you take your antihypertensive medicine yesterday?

Item 6. When you feel like your antihypertensive is under control, do you sometimes stop taking your medicine?

Item 7. Taking medication every day is a real inconvenience for some people. Do you ever feel hassled about sticking to your antihypertensive treatment plan?

Item 8. How often do you have difficulty remembering to take all your medications?

Never, rarely, sometimes, usually, all the time.
